# Constitutive expression of active microbial transglutaminase in *Escherichia coli* and comparative characterization to a known variant

**DOI:** 10.1186/s12896-017-0339-4

**Published:** 2017-02-28

**Authors:** Gabe Javitt, Zohar Ben-Barak-Zelas, Moran Jerabek-Willemsen, Ayelet Fishman

**Affiliations:** 1 Department of Biotechnology and Food Engineering, Technion-Israel Institute of Technology, Haifa, 3200003 Israel; 2 NanoTemper Technologies GmbH Flößergasse 4, 81369 Munich, Germany

**Keywords:** Microbial transglutaminase, Organic solvents, Thermostable, Differential scanning fluorimeter

## Abstract

**Background:**

Microbial transglutaminase (mTG) is a robust enzyme catalyzing the formation of an isopeptide bond between glutamine and lysine residues. It has found use in food applications, pharmaceuticals, textiles, and biomedicine. Overexpression of soluble and active mTG in *E. coli* has been limited due to improper protein folding and requirement for proteolytic cleavage of the pro-domain. Furthermore, to integrate mTG more fully industrially and academically, thermostable and solvent-stable variants may be imperative.

**Results:**

A novel expression system constitutively producing active mTG was designed. Wild-type (WT) mTG and a S2P variant had similar expression levels, comparable to previous studies. Kinetic constants were determined by a glutamate dehydrogenase-coupled assay, and the S2P variant showed an increased affinity and a doubled enzyme efficiency towards Z-Gln-Gly. The melting temperature (T_m_) of the WT was determined by intrinsic fluorescence measurements to be 55.8 ± 0.1 °C and of the S2P variant to be 56.3 ± 0.4 °C and 45.5 ± 0.1 °C, showing a moderately different thermostability profile. Stability in water miscible organic solvents was determined for both the WT and S2P variant. Of the solvents tested, incubation of mTG in isopropanol for 24 h at 4 °C showed the strongest stabilizing effect with mTG retaining 61 and 72% activity for WT and S2P respectively in 70% isopropanol. Both enzymes also showed an increased initial activity in the presence of organic solvents with the highest activity increase in 40% DMSO. Nevertheless, both enzymes were inactivated in 70% of all organic solvents tested.

**Conclusions:**

A constitutive expression system of active mTG in *E. coli* without downstream proteolytic cleavage processing was used for overexpression and characterization. High throughput techniques for testing thermostability and kinetics were useful in streamlining analysis and could be used in the future for quickly identifying beneficial mutants. Hitherto untested thermostability and stability of mTG in organic solvents was evaluated, which can pave the way for use of the enzyme in novel applications and processes.

## Background

Transglutaminase (EC 2.3.2.13, protein-glutamine gamma-glutamyltransferase, TG) catalyzes the acyl transfer reaction between γ-carboxyamide groups (acyl donor) and primary amines (acyl acceptor). In proteins, it is able to crosslink the γ-carboxyamide of glutamine and the primary ε-amine in lysine. Studies show that other acyl acceptors can be used, such as esterified threonine, serine, cysteine, and tryptophan [1]. In humans, TG is involved in formation of clots, wound repair, epidermal keratinization, and membrane curing [2], but in plants it functions in structural modification of specific protein substrates and programmed cell death [3].

In Nature, TG are prevalent in all life domains including plants [3], mammals [4], and microorganisms [2]. Microbial TG (mTG) are distinct from mammalian and botanical sources being Ca^+2^ independent, smaller in size, and composed of a single domain instead of multi-domains. The active domain in all sources contains a catalytic triad composed of Cys, His and Asp, although the configuration differences suggest a convergent evolution model [5–7]. mTG are robust enzymes retaining activity over a wide range of pH and temperature [8]. mTG from *Streptomyces mobaraensis*, the only commercially available enzyme [2], is expressed extracellularly as a zymogen containing an N-terminal pro-domain required for proper folding that covers the active site along with an extracellular protease that cleaves off the pro-domain thus producing the mature active enzyme [5].

mTG has been accepted as Generally Recognized as Safe (GRAS) by the FDA since 1998, and has subsequently been widely used in the food and textile industries for modifying proteins [9, 10]. It is commonly used in the restructuring of meat products, thickening of yoghurts, coagulation alternatives for tofu, and more recently in molecular gastronomy [9, 11]. In textiles it has been used successfully to improve shrink resistance, tensile strength, and color retention, and to crosslink proteins into leather to fill voids [10]. Increasingly it is also being studied in the biomedical industry for site-specific PEGylation of proteins, antibody modifications, and crosslinking hydrogels for drug delivery [1].

Commercial production of mTG has focused mainly on wild-type *Streptomyces* spp. yielding extracellular enzyme with activity up to 6.0 U/ml which is considered low industrially [12]. Furthermore, it is not a convenient system for producing improved variants with tailored properties. Other expression systems using genetically modified microorganisms have been studied, including *Corynebacterium* spp.*, Escherichia coli,* and *Candida boidinii*, although these were unable to out-produce the native *Streptomyces* spp. [12]. Therefore, generation of high yield recombinant mTG in *E. coli* would be useful in biotechnology being a widely studied system with availability of high level expression vectors and strains [13]. *E. coli* is already used for production of nearly 30% of therapeutic recombinant proteins [13], and is a commonly used platform for enzyme engineering. Moreover, recombinant overexpression of foreign proteins in *E. coli* can constitute over 45% of total protein produced, highlighting the potential [14].

Initial studies on cloning and expression of soluble *Streptomyces* spp. mTG in *E. coli* had limited success due to the formation of inclusion bodies or low yields [12]. Recent studies have been able to produce high levels of chemically renatured mTG fusion proteins from inclusion bodies [15]. Other production systems involve a secondary in-vitro protease activation step to remove the pro-domain [16], or by introduction of a protease recognition site, and co-expression of the protease with the mTG [17]. An elegant approach reported by Liu et al. [18] has been the cloning of a polycistronic gene with the pro-domain required for proper folding and the mature enzyme after cleavage expressed by the same T7 promoter noncovalently one following the other. The expressed products were secreted into the periplasm for proper folding and protease protection. This method avoids downstream proteolytic cleavage, simplifying the production of active mTG.

In recent years there has been a strong push for industrial processes to become “greener”, which includes increased efficiency, increased safety of the process and of the final product, and benign to the environment [19]. Integration of enzymatic biocatalysts into industrial processes has proven itself as one way to achieve these goals. Enzymes are able to work in mild conditions while still achieving high activity, and have inherent selectivity, thus increasing efficiency and decreasing waste [20, 21]. On the other hand, there is an incentive to increase the thermostability and solvent stability of functional enzymes. Higher temperatures enhance chemical reaction rates, increase solubility, and decrease contaminant risk by microorganisms, although most enzymes evolved in mesophilic conditions, such as mTG, are prone to denaturation at temperatures used industrially [22]. Organic solvents are commonly used in industrial applications and solvent-stability of enzymes holds several advantages, such as increased solubility of hydrophobic substrates, protection from undesired hydrolytic reactions, and shifting of the thermodynamic equilibriums towards synthesis [23].

The present work describes an improved strategy for cloning and constitutive expression of soluble active mTG from *Streptomyces mobaraensis* in *E. coli*, and the subsequent purification and characterization of the enzyme*.* Thermal and organic solvent stability of the wild-type (WT) and variant S2P were studied for the first time while the kinetic constants were measured using a modified version of a recently reported assay allowing a rapid comparison between the enzymes. Additionally, the S2P enzyme was tested for crosslinking soy protein isolate to compare it to the WT mTG in an actual food application.

## Results and discussion

### Cloning, expression, and purification of a recombinant mTG in E. coli

**Fig. 1:**
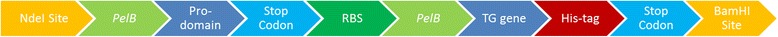
Synthetic mTG construct used for transformation of *E. coli* BL21 (DE3) cells. The construct consists of restriction enzyme sites, the pro-domain required for folding and the mTG gene separately expressed. Each one has a preceding *PelB* sequence to direct periplasm secretion, whereupon folding will occur in the periplasm. Another ribosome binding site (RBS) precedes the mTG gene to ensure proper translation

The main goal of the present work was to use *E. coli* as an efficient expression system for the production of an active and soluble mTG from *Streptomyces mobaraensis.* Of the methods described in the literature and highlighted in the Background, that of Liu et al. [18] comprising a polycistronic gene with the pro-domain and the active gene was the most promising starting point. The expressed products from this construct were secreted into the periplasm. Expression of recombinant proteins in the periplasm facilitates the successful folding of enzymes, reducing the formation of inclusion bodies [24]. Additionally, periplasm production may help reduce enzyme degradation by cytosolic proteases [25]. The synthetic operon construct (based on GenBank: KX775947) consisted of two parts: i) a gene encoding the pro-domain crucial for proper folding of the enzyme and ii) the gene encoding the mTG thermostable variant S2P [26], with a C-terminal His-tag. Each part was paired with a preceding *PelB* secretory sequence (Fig. 1). Primers which included the NdeI and BamHI restriction sites were used to amplify the synthetic construct, and clone it into a pET9a vector for transformation into *E. coli* BL21 (DE3) cells. The sequence was confirmed by sequence analysis after transformation. The change from the pET22b used by the original group, to a constitutive vector pET9a helped streamline the expression, negating the addition of an inducer following an incubation period, while still achieving comparable levels of expression.

In order to compare the mutant to the WT enzyme, primers were designed with the P2S site reversion mutation. The plasmid was extracted and amplified using the primers, after which DpnI enzyme was used to digest the template plasmid. Separately, the S2P construct was transferred into a pET22b vector for expression in *E. coli* BL21 (DE3) cells, to compare the constitutive expression of the pET9a with the inducible expression of pET22b used by the original group [18].

The cells were grown in separate batches, disrupted, and the mTGs were purified by affinity chromatography on a nickel column. The resulting purified enzymes were compared on an SDS-PAGE gel, and by spectroscopy for purity and concentration. All expression vectors led to comparable enzyme purity and concentrations of 120 mg/L or 1 U/ml.

This expression system allows production of active mTG without requiring any further processing steps such as renaturing from inclusion bodies or cleavage of a pro-enzyme to yield the active enzyme [15–17, 27]. Such methods require time, while these changes allow for a faster expression which more closely resembles the current industrial production which purifies active mTG from *Streptomyces* without any activation step. An advantage with this expression system in the containment of the active enzyme intracellularly, reducing the working volume for extraction as opposed to an extracellular extraction. In addition, the constitutive expression system shows that accumulation of active mTG in the periplasm is not completely toxic to *E. coli* as previously thought [15, 28].

A further increase in expression levels may be possible by co-expressing periplasmic secretory proteins, since secretion into the periplasm is frequently the limiting factor for production [24]. Another strategy could include using periplasm protease deficient *E. coli* strains which would reduce enzyme degradation [24].

In order to compare the purified enzyme produced using the constitutive expression system to previous reports, WT and S2P variant were biochemically characterized. Furthermore, thermostability and organic solvent stability were assessed.

### Kinetic properties of WT and S2P mutant

The S2P variant was produced by random mutagenesis of the WT enzyme, and found to be more thermostable - able to withstand incubation at 60 °C - and more active than the WT [26, 29]. The kinetic constants of WT mTG and variant S2P were determined using the coupled enzyme reaction proposed by Oteng-Pabi and Keillor [30]. The enzymatic transamidation reaction between a γ-glutamyl donor (Z-Gln-Gly) and hydroxylamine releasing ammonia was coupled to the glutamate dehydrogenase (GDH)-catalyzed reductive amination of α-ketoglutarate (α-KG). The activity of GDH is dependent on NADH as a co-factor, whose disappearance can be monitored at 340 nm. NADH concentration was calculated based on a calibration curve, which in turn was used to calculate activity of mTG. The enzymes analyzed were the WT and the S2P variant purified from *E. coli* harboring the pET9a vector. The activity profile followed Michaelis-Menten kinetics, and the results are presented in Table 1.

**Table 1:** Kinetic constants of WT and S2P variant measured by the mTG-GDH coupled enzyme method^a^

Variant	K_m_ (mM)	k_cat_ (1/s)	k_cat_/K_m_ (1/s⋅mM)
WT	11.6 ± 0.3	0.850 ± 0.007	0.073 ± 0.002
S2P	4.2 ± 0.1	0.60 ± 0.01	0.141 ± 0.002

^a^Results are the average of three independent experiments with 5 assay repetitions in each experiment

As evident from Table 1, the K_m_ value was 3-fold lower for the mutant as compared to the WT, indicating a higher affinity for the substrate. Conversely, the turnover number was higher for the WT enzyme, although the enzymatic efficiency was 2-fold higher for the mutant.

The kinetic constants for the S2P variant had not been determined beforehand, although it had been found to be more stable and active [26, 29]. The assay used in this research [30], is continuous and efficient, allowing rapid comparison between different variants. The results show (Table 1) that variant S2P has a statistically significant lower K_m_ value than WT (4.2 mM and 11.6 mM respectively, *p* = 0.002). These values deviate from the literature values for WT mTG (40–55 mM) [5, 30, 31], although different assay conditions or different substrates can account for these discrepancies. Oteng-Pabi and Keillor who developed the continuous assay [30], used Gly-OMe instead of hydroxylamine as the acyl acceptor, which could result in different kinetic constants than obtained here. In addition, their mTG was activated by incubation with trypsin, which leaves a FRAP peptide from the pro-domain on the C terminal of the mature mTG [32], and is therefore a slightly different enzyme. Other groups [5, 31] who determined kinetic constants used the standard hydroxamate procedure [30, 33], which is an endpoint assay rather than a continuous method, which determines the rate of reaction based on a single point instead of multiple points. Additionally, Zhang and co-workers used a native mTG without a His-tag, which again, may alter the kinetic properties to some extent [31]. Finally, the coupled reaction is performed at pH 7.2, while the other groups used pH of 6.0 as per the standard hydroxamate assay.

Nonetheless, these results, coupled with the variants’ increased enzyme efficiency (k_cat_/K_m_) of 0.14 1/s⋅mM versus 0.07 1/s⋅mM for the WT, support the increased activity found previously [29]. However, the S2P’s decreased turnover number (k_cat_) supports the notion of a reduced activity at higher concentrations. The difference between these results and the previous findings for the S2P having a higher specific activity than the WT enzyme may be explained by the hydroxamate activity assay protocol being done at a substrate concentration below the V_max_ [30, 33]. Using a lower substrate concentration, the variant, having a lower K_m_, would show increased activity compared to the WT, although at higher substrate concentrations those differences disappear. Therefore, although the S2P variant does show an increased affinity and efficiency, its benefit is only seen at lower substrate concentrations, and further highly active mutant characterization should also focus on activity assays at higher substrate concentrations to ensure enzyme saturation.

### Thermostability of WT and S2P mTG

**Fig. 2:**
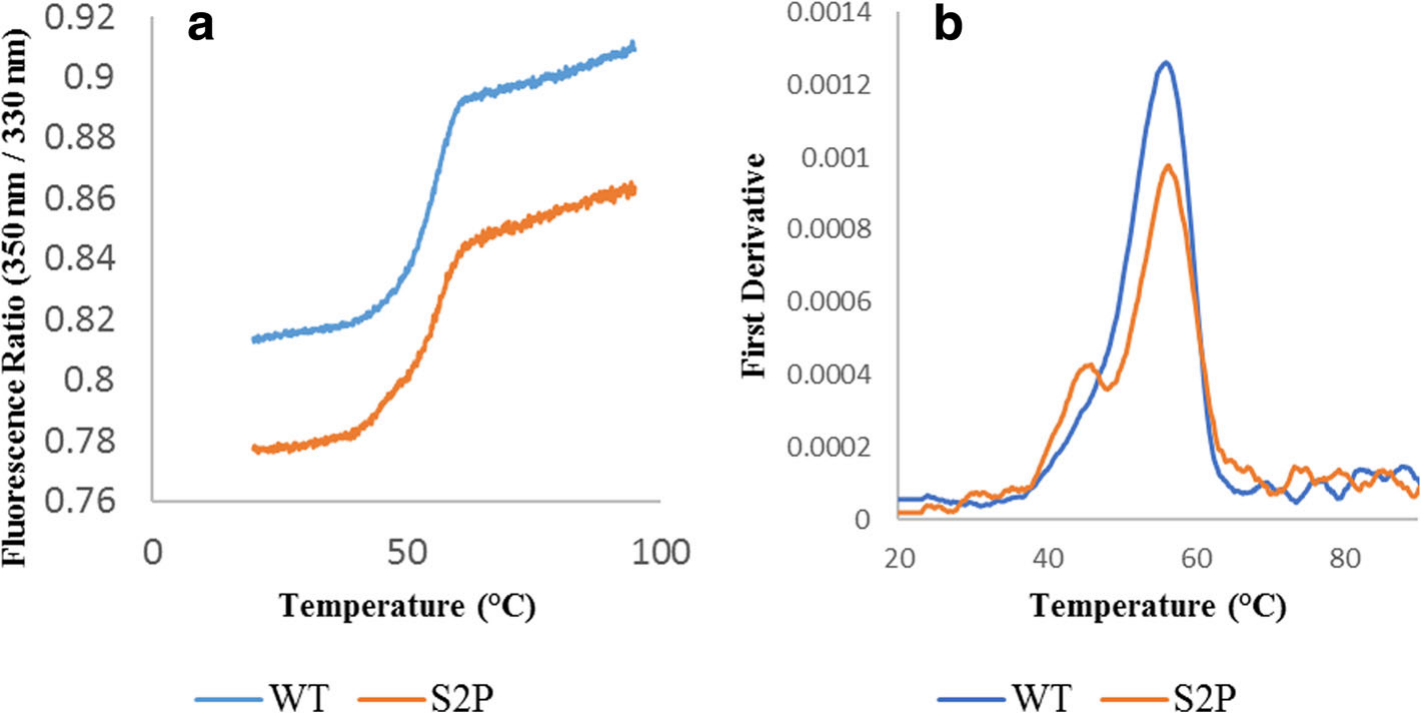
Melting scan of the fluorescence ratio versus temperature (**a**) and first derivate versus temperature (**b**) of WT and S2P variant. Results represent an average of duplicates

For industrial applications, a highly thermostable mTG could be beneficial. Variant S2P was found to have a significantly increased residual activity after incubation at 60 °C compared to the WT enzyme [26]. Thermal stability of the enzyme itself as opposed to residual activity after incubation was tested here for the first time using a differential scanning fluorimeter (nanoDSF) (Fig. 2). This instrument measures the change in fluorescence of tyrosine and tryptophan amino acids upon protein unfolding at increasing temperatures [34]. In the fluorescence ratio, and in the first derivative, there are two inflection points and melting temperatures (T_m_) for the S2P variant while the WT has only one T_m_. Height of peaks is related to protein concentration and not to thermostability. The peak of the S2P variant was at a higher temperature than the WT enzyme, and showed an additional peak at a lower temperature. Results of the DSF measurement are summarized in Table 2 based on the built-in software.

**Table 2:** Results of denaturation temperatures of WT and S2P mTG enzymes as determined by nanoDSF^a^

Variant	T_m_ 1 (°C)	T_m_ 2 (°C)
WT	55.8 ± 0.1	-
S2P	56.3 ± 0.4	45.5 ± 0.1

^a^ Results are the average of duplicates

As can be seen from Fig. 2 and Table 2, the mutant unfolded at a slightly higher temperature (56.3 °C vs. 55.8 °C) indicating improved thermostability although not statistically significant. Additionally, a second T_m_ was observed at 45.5 °C for the variant but not for the WT enzyme. Position two is important for stabilization of two loops in mTG [35], and the substitution of the polar serine to the apolar proline may diminish these loop interaction and cause a secondary melting temperature in this region. The nanoDSF may therefore offer a fast high-throughput test for thermostability and help explain conformational changes of mutations previously accessible by crystal structure determination only. Furthermore, the determination of the WT T_m_ allows for future comparison to other thermostable variants.

### Stability in organic solvents

Enzymatic stability in organic solvents is an important property in applied biocatalysis and for some industrial applications [36]. In the pharmaceutical industry for example, there is a high use of organic solvents for the entire process of drug production including solubilizing, extracting, crystallization, and reacting with the drug [35]. Moreover, in the textile industry, organic solvents are important for dyeing leather [36], and for finishing, scouring, desizing, and coating of fabrics [37]. Use of mTG has been studied for some processes in the pharmaceutical industry [38, 39] and textile industry [10, 40, 41], although increasing the stability in organic solvents may allow more widespread acceptance and integration.

**Fig. 3:**
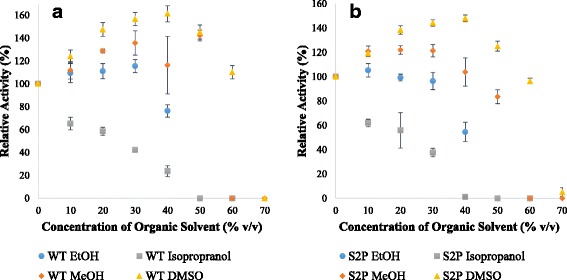
Effect of organic solvents on residual activity of mTG after incubation for 24 h at 4 °C. WT (**a**) and S2P (**b**) were assayed for activity after incubation using the standard hydroxamate assay. Activity of mTG incubated in 50 mM sodium phosphate buffer pH 8 was designated as 100% activity, and used for comparison. Results represent an average of duplicates

**Fig. 4:**
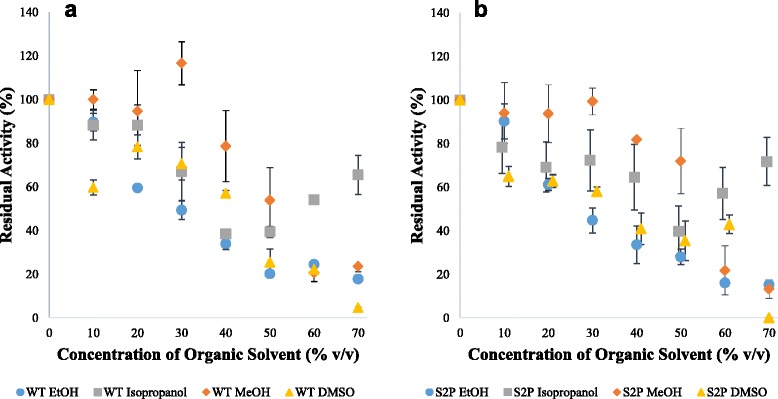
Effect of organic solvents on the activity of mTG; WT (**a**) and S2P (**b**). The standard hydroxamate activity assay was performed with the addition of varying concentrations of organic solvents. 100% activity was defined as the activity in 50 mM sodium phosphate buffer pH 8. Results represent an average of triplicates

In order to characterize mTG stability in organic solvents two tests were performed. The first was incubation overnight at 4 °C in the presence of organic solvents followed by measurement of the residual activity (Fig. 3). This temperature was chosen to minimize mTG self-polymerization. The second was performing the activity assay in the presence of organic solvents and comparing them to the activity in buffer (Fig. 4). The organic solvents used included water-miscible methanol (MeOH), ethanol (EtOH), isopropanol, and dimethyl sulfoxide (DMSO).

Enzyme stability differed among the organic solvents tested. The residual activity was stable after incubation in low-range concentrations of methanol and isopropanol (0–30%), while there was a decrease in activity in ethanol and DMSO starting from 10%. mTG seems particularly stable in isopropanol, retaining ~61 and ~72% activity for WT and S2P respectively in 70% isopropanol (Fig. 3). The mutant enzyme showed comparable stability as the WT in most organic solvents, although had a higher residual activity in isopropanol.

As opposed to 24-h incubation, activity in the presence of organic solvents had a different stability profile (Fig. 4). The activity of WT and S2P was highest in DMSO followed by MeOH, EtOH, and isopropanol. The S2P mutation also seemed to have a negligible influence on the activity in most organic solvents, although activity in isopropanol was reduced. The activity in the presence of 10–30% organic solvents also increased, with the highest activity seen in 40% DMSO. Similar results have been previously shown for tyrosinase from *Bacillus megaterium* [42]. At 70% concentration, activity in all tested organic solvents ceased, indicating a maximum usable concentration, at least for the solvents evaluated.

All solvents tested were polar, more capable of penetrating into the enzyme core and inducing conformational changes than non-polar solvents [43]. The increased activity following incubation in organic solvents may be linked to induction of different stable conformations, while the increased activity in organic solvents may be linked to a looser conformation which could ease entrance of substrates into the catalytic pocket. Loss of activity at higher concentrations is likely caused by denaturation of the enzyme [44], although the increased residual activity in high concentrations of isopropanol is unexpected which warrants further study and may make it an excellent storage medium for mTG. Another possibility is that the organic solvents improve the selectivity of the enzyme, as has been shown with other enzymes [42, 45]. As noted previously all of the tested organic solvents are commonly used in the pharmaceutical industry [35], and by knowing the stability of mTG in various solvents, it might be possible to combine current drug production with novel applications of mTG such as antibody drug conjugates [39].

### Crosslinking of soy protein isolate (SPI)

**Fig. 5:**
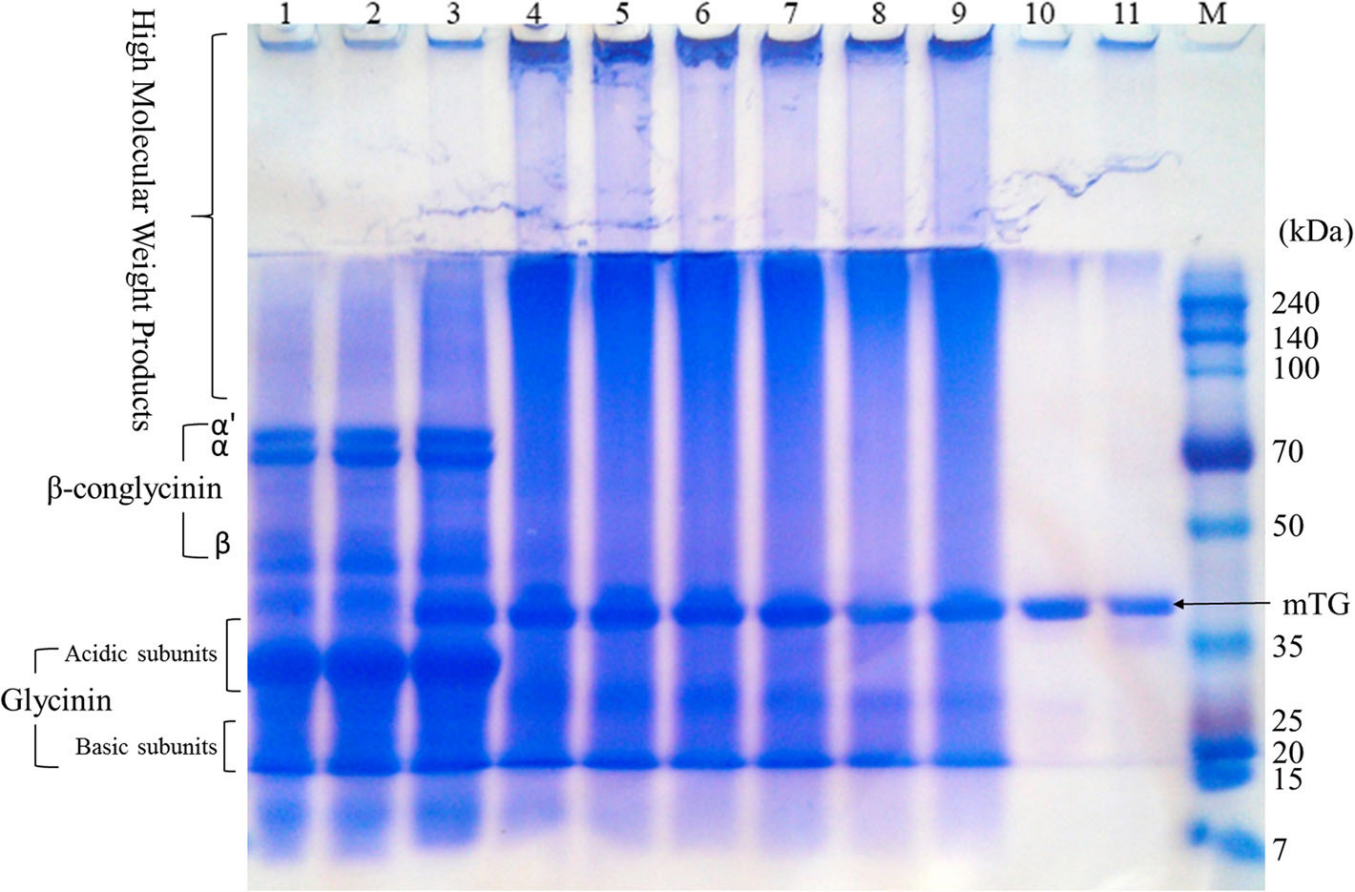
SPI crosslinking by S2P mTG for 4 h. From left to right: lanes 1–2 present SPI alone at 0 min and at 4 h; lanes 3–9 show SPI with mTG at 0 min, 15 min, 30 min, 45 min, 1 h, 2 h, 4 h, respectively; lanes 10–11 present mTG alone at 0 min and at 4 h; M is the protein size ladder. The SDS-PAGE was run using 15 μl per well from each fraction

mTG is mostly used in food applications for modulating texture and rheological properties [27, 46–48]. The WT mTG had been shown previously to crosslink soy protein isolate (SPI) [49]. The S2P variant was therefore tested here for its ability to crosslink SPI, ensuring the mutation did not hinder the ability to crosslink native proteins, and reconfirming the validity of the activity assay. S2P was incubated with a 1% solution of SPI at a 1:50 gr/gr concentration of mTG to SPI for 4 h, and run on an SDS-PAGE (Fig. 5).

The crosslinking was evidenced by production of high molecular weight products at the top of the stacking gel, and the top of the separating gel, in addition to the disappearance of the β-conglycinin and acidic subunit glycinin protein bands. In addition, the control of mTG alone and SPI alone confirm that the crosslinking was done by the mTG and not spontaneously, and that the mTG is stable over 4 h. The activity of S2P was consistent with that of WT mTG in its ability to crosslink β-conglycinin and acidic subunits of glycinin but not the basic subunits of glycinin [50]. These findings, in addition to the kinetic results highlight that although S2P mTG has a lower K_m_ it is still limited to specific substrates.

## Conclusions

This is the first time that active and soluble mTG has been overexpressed in a constitutive vector in *E. coli* without requiring any downstream processing as proteolytic digestion of renaturation from inclusion bodies. From this expression system, a high-throughput continuous kinetic analysis was able to quantify the improvement of a known variant. The thermostability characteristics were also determined in a novel fashion using intrinsic fluorescence measurement, perhaps enabling a faster alternative to current assays. Finally, the enzymes were evaluated for activity in organic solvents, creating a benchmark for future studies and integration into novel pathways.

## Methods

### Chemicals

Z-Gln-Gly as a γ-glutamyl donor substrate was purchased from Sigma-Aldrich (Rehovot, Israel), and hydroxylamine was purchased from Spectrum Chemicals (New Jersey, USA). Organic solvents were of analytical grade and purchased from Gadot Biochemical Industries LTD. (Haifa, Israel). All other buffers and chemicals were of analytical grade. SPI was kindly provided by CHS (Minnesota, USA).

### Design and production of plasmid vectors

The engineered plasmid was based on the S2P transglutaminase produced by random mutagenesis by Pietzsch et al. [29], using the construct of the pro-domain and mature transglutaminase expressed one following the other [18]. The construct consisted of a NdeI restriction enzyme site followed by a *pelB* sequence before the pro-domain sequence with a stop codon, a spacer of 54 bps containing another RBS (5’GGATAACAATTCCCCTCTAGAAATAATTTTGTTTAACTTTAAGAAGGAGATATA3’) and then another *pelB* sequence followed by the mature transglutaminase sequence with a hexa histidine-tag for purification and finally a BamHI restriction enzyme site (Fig. 1). The construct was optimized and synthesized by GenScript (NJ, USA), and amplified using a PCR reaction with forward primer 5’CCCAAACATATGAAATACCTGCTGCCG3’ and reverse primer 5’GTGTGTGGATCCTCAGTGGTGGTGGTG3’ synthesized by HyLabs (Rehovot, Israel) with a Phusion DNA polymerase (Thermo Fisher; Massachusetts, USA) in a 50 μl reaction using a thermocycler (Tpersonal; Biometra, Göttingen, Germany). The PCR program had an initial denaturation step for 30 s at 98 °C, followed by 30 cycles of 30 s at 98 °C, 30 s at 73 °C and 1 min at 72 °C followed by a final elongation step of 10 min at 72 °C. The PCR product was purified using a QIAquick PCR Purification Kit (Qiagen; Hilden, Germany). The PCR product and the vector pET9a (Novagen; Darmstadt, Germany) were both digested with NdeI and BamHI, and were then ligated with T4 DNA ligase (Promega; Madison, Wisc., USA). The ligated plasmid was transformed into competent *E. coli* BL21 (DE3) using a GeneZapper (Bio-Rad; Hercules, Calif., USA), and grown on LB agar plates containing kanamycin (25 μg/ml). Plasmids from colonies signifying successful transformation, were extracted using a plasmid miniprep kit (Qiagen; Hilden, Germany), and sequenced for verification.

The WT plasmid was produced by reverse mutation of the synthesized S2P variant using a primer containing the WT serine in position 2. The forward primer used was 5’ GCG ATG GCG ATG GAC TCG GAT GAT CGC GTG ACC 3’, and reverse primer used was 5’ GGT CAC GCG ATC ATC CGA GTC CAT CGC CAT CGC 3’. Taq polymerase (EurX; Gdańsk, Poland) was incubated in a thermocycler (Tpersonal; Biometra, Göttingen, Germany) with the purified pET9a plasmid containing the synthesized gene. The PCR program had an initial denaturation step for 30 s at 94 °C, then 30 cycles of 10 s at 94 °C 30 s at 75 °C and 6 min at 74 °C, followed by a final elongation step for 10 min at 74 °C. The template plasmid was digested for 18 h at 37 °C with a Dpn1 enzyme (NEB; Massachusetts, USA). The resulting plasmid was prepped, used for transformation, and verified by sequencing in the same manner as the S2P mutant plasmid. To evaluate the benefit of using pET9a instead of pET22b originally used, the TG gene was transferred from the pET9a to the pET22b using standard methods.

### Expression and purification of mTG

Transformed *E. coli* were grown on LB plates containing kanamycin overnight at 37 °C and seeded into 500 ml Terrific Broth containing 25 μg/mL kanamycin. After incubation for 60 h at 25 °C, the samples were centrifuged at 4,000 rpm for 15 min and 4 °C. The resulting pellet was resuspended in binding buffer (500 mM NaCl, 30 mM imidazole) and thrice passed through a homogenizer (EmulsiFlex-C3, AVESTIN Inc.; Ottawa, Canada) at 1,500 bar. The cell debris was removed from the supernatant by centrifuging twice at 16,000 g for 20 min at 15 °C, whereas the supernatant was applied to a nickel affinity column (HisTrap 5 ml; GE Healthcare; Buckinghamshire, United Kingdom) primed with the binding buffer.

Elution was performed using a linear gradient with an appropriate buffer (500 mM NaCl, 500 mM imidazole). The fractions containing TG were collected and dialyzed against a phosphate buffer at 4 °C (50 mM sodium phosphate buffer pH 8). Protein concentration was calculated with a NanoDrop 2000 (Thermo Fisher Scientific Inc. Massachusetts, USA) using a molar extinction coefficient of 71,850 1/M⋅cm and molecular weight of 39.07 kDa, calculated using the ProtParam (Swiss Institute of Bioinformatics) online software.

### SDS-PAGE analysis

SDS-PAGE was performed according to the Laemmli discontinuous buffer system [51] using a 4% stacking gel and 12% separating gel. The samples mixed with sample buffer were heated for 10 min at 95 °C. After running, the gel was stained with 0.25% Coomassie brilliant blue (R-250) solution (40% Coomassie, 50% ethanol, 10% acetic acid), and destained in a destain solution (20% methanol, 10% acetic acid, 60% dH_2_O (v/v/v)).

### Hydroxamate activity assay

mTG activity was determined using the hydroxamate assay at pH 6.0 and 37 °C [30]. Briefly, 200 μL substrate mixture comprising 66.5 mM Z-Gln-Gly, 440 mM hydroxylamine, 2.2 mM EDTA, in 445 mM Tris-acetate pH 6.0 were incubated at 37 °C for 5 min. An mTG solution (50 μL) in 50 mM sodium phosphate buffer pH 8 was added. After 10 min of reaction, 250 μL of assay reagent (2 M FeCl_3_, 0.3 M Trichloroacetate, 0.8 M HCl) was added and centrifuged for 5 min at 13,300 rpm. The supernatant was transferred to a microplate, and the resulting color was measured at 525 nm. Activity was calculated by comparing to a calibration curve using L-glutamic acid γ-monohydroxamate from 0 to 5 mM. One unit was defined as the formation of 1 μmol product per minute.

For determination of thermostability, 0.5 mg/ml of WT and S2P enzymes were loaded into the capillary tubes of a Prometheus NT.48 nanoDSF (NanoTemper Technologies GmbH; Germany), and heated at a rate of 2 °C/min. Melting temperatures (Tm) were calculated by the NT Melting Control software. The T_m_ was determined by fitting the experimental data to a polynomial function, in which the slope maxima are indicated by the peak of its first derivative.

### Kinetic constant analysis via a coupled enzyme assay

Kinetic constants were calculated using the coupled enzyme assay [30], in which the ammonia release from mTG activity is coupled to the uptake by glutamate dehydrogenase (GDH). The ammonia, which is the byproduct of mTG catalyzed transamidation reaction between Z-Gln-Gly and hydroxylamine, was used as a substrate for GDH.

The activity of GDH is dependent on NADH as a co-factor, whose disappearance can be monitored at 340 nm. 180 μL reaction mixture per microplate well contained 0.2 M MOPS pH 7.2, 1 mM EDTA, 10 mM α-KG, 2U GDH, 10 mM hydroxylamine, 0.5 mM NADH, and 0.25-50 mM Z-Gln-Gly final concentration. The plate was equilibrated for 5 min at 37 °C prior to addition of 20 μl mTG (1 U) or water instead of mTG as a blank. The concentration of NADH was calculated based on a calibration curve. Reactions were carried out in a 96 multi-well plate, and the absorbance was recorded in a BioTek EON plate reader (Vermont, USA). Kinetic constants were calculated using the Enzyme Kinetics toolbox in SigmaPlot 12 (Systat Software Inc.).

### Stability in organic solvents

The enzymes were tested for stability in organic solvents based on an activity assay in the presence of organic solvents, and the activity after incubation in organic solvents for 24 h at 4 °C. The relative activity in the presence of organic solvents was based on the hydroxamate activity assay [29]. The organic solvents were added in addition to the substrate buffer before the addition of the enzyme, to produce the desired final concentration. Activity was corrected for the increased volume due to the organic solvent, and compared to a blank containing water instead of enzyme.

Residual activity after incubation in organic solvents was also calculated based on the hydroxamate assay. 2.5 U/ml mTG was incubated in different concentrations of organic solvents (10–80%) at 4 °C for 24 h. After incubation, 50 μl of mTG in organic solvents was used directly in the standard hydroxamate assay. Activity was compared to a blank of water instead of mTG. Activity of mTG incubated in 50 mM sodium phosphate buffer pH 8 at 4 °C for 24 h was set as 100%, and used as the benchmark for the other activities.

### Crosslinking of soy protein isolate (SPI)

1% SPI solution (w/v) was suspended in 50 mM SPB pH 7.4 and stirred for 12 h at 4 °C. The solution was centrifuged at 13,300 rpm for 5 min to remove non soluble protein, and the supernatant was mixed with mTG to obtain a 1:50 w/w solution. The solution was incubated at 37 °C in an orbital shaker (TU-400 MRC; Holon, Israel) at 200 rpm for 4 h. Two controls were incubated in the same conditions consisting of either mTG in water or SPI in water. Samples from the reactions were taken at 0, 15, 30, 45, 60, 120, and 240 min and mixed with SDS-PAGE sample buffer to stop the reaction and prepare the fractions for SDS-PAGE gel visualization.

### Statistical analysis

The significance of differences between means was determined by paired sample Student’s *t* test. The level of significance used was 95% or higher.

## Abbreviations

DMSO: Dimethyl sulfoxide
DSF: Differential scanning fluorimeter
EtOH: Ethanol
GDH: Glutamate dehydrogenase
GRAS: Generally recognized as safe
MeOH: Methanol
mTG: Microbial transglutaminase
NADH: Nicotinamide adenine dinucleotide
WT: Wild type

## References

[1] Gundersen MT, Keillor JW, Pelletier JN (2014). Microbial transglutaminase displays broad acyl-acceptor substrate specificity. Appl Microbiol Biotechnol.

[2] Martins IM, Matos M, Costa R, Silva F, Pascoal A, Estevinho LM, Choupina AB (2014). Transglutaminases: recent achievements and new sources. Appl Microbiol Biotechnol.

[3] Serafini-Fracassini D, Del Duca S (2008). Transglutaminases: widespread cross-linking enzymes in plants. Ann Bot.

[4] Eckert RL, Kaartinen MT, Nurminskaya M, Belkin AM, Colak G, Johnson GV, Mehta K (2014). Transglutaminase regulation of cell function. Physiol Rev.

[5] Yang MT, Chang CH, Wang JM, Wu TK, Wang YK, Chang CY, Li TT (2011). Crystal structure and inhibition studies of transglutaminase from *Streptomyces mobaraense*. J Biol Chem.

[6] Schroeder V, Kohler HP (2016). Factor XIII: structure and function. Semin Thromb Hemost.

[7] Aloisi I, Cai G, Serafini-Fracassini D, Del Duca S. Transglutaminase as polyamine mediator in plant growth and differentiation**.** Amino Acids. 2016;48:2467–78. 10.1007/s00726-016-2235-y 27101214

[8] Yokoyama K, Nio N, Kikuchi Y (2004). Properties and applications of microbial transglutaminase. Appl Microbiol Biotechnol.

[9] Gaspar AL, de Goes-Favoni SP (2015). Action of microbial transglutaminase (MTGase) in the modification of food proteins: a review. Food Chem.

[10] Tesfaw A, Assefa F (2014). Applications of transglutaminase in textile, wool, and leather processing. Int J Tex Sci.

[11] Ruiz J, Calvarro J, Sánchez del Pulgar J, Roldán M (2013). Science and technology for new culinary techniques. J Culin Sci Technol.

[12] Zhang D, Zhu Y, Chen J (2010). Microbial transglutaminase production: understanding the mechanism. Biotechnol Genet Eng Rev.

[13] Huang CJ, Lin H, Yang X (2012). Industrial production of recombinant therapeutics in *Escherichia coli* and its recent advancements. J Ind Microbiol Biotechnol.

[14] Rosano GL, Ceccarelli EA (2014). Recombinant protein expression in *Escherichia coli*: advances and challenges. Front Microbiol.

[15] Salis B, Spinetti G, Scaramuzza S, Bossi M, Saccani Jotti G, Tonon G, Crobu D, Schrepfer R (2015). High-level expression of a recombinant active microbial transglutaminase in *Escherichia coli*. BMC Biotechnol.

[16] Sommer C, Hertel TC, Schmelzer CE, Pietzsch M (2012). Investigations on the activation of recombinant microbial pro-transglutaminase: in contrast to proteinase K, dispase removes the histidine-tag. Amino Acids.

[17] Rickert M, Strop P, Lui V, Melton-Witt J, Farias SE, Foletti D, Shelton D, Pons J, Rajpal A (2016). Production of soluble and active microbial transglutaminase in *Escherichia coli* for site-specific antibody drug conjugation. Protein Sci.

[18] Liu S, Zhang DX, Wang M, Cui WJ, Chen KK, Du GC, Chen J, Zhou ZM (2011). The order of expression is a key factor in the production of active transglutaminase in *Escherichia coli* by co-expression with its pro-peptide. Microb Cell Fact.

[19] Anastas P, Eghbali N (2010). Green chemistry: principles and practice. Chem Soc Rev.

[20] Sheldon RA, van Pelt S (2013). Enzyme immobilisation in biocatalysis: why, what and how. Chem Soc Rev.

[21] Shoda S, Uyama H, Kadokawa J, Kimura S, Kobayashi S (2016). Enzymes as green catalysts for precision macromolecular synthesis. Chem Rev.

[22] Yu H, Huang H (2014). Engineering proteins for thermostability through rigidifying flexible sites. Biotechnol Adv.

[23] Stepankova V, Bidmanova S, Koudelakova T, Prokop Z, Chaloupkova R, Damborsky J (2013). Strategies for stabilization of enzymes in organic solvents. ACS Catal.

[24] Yoon SH, Kim SK, Kim JF (2010). Secretory production of recombinant proteins in *Escherichia coli*. Recent Pat Biotechnol.

[25] Choi JH, Lee SY (2004). Secretory and extracellular production of recombinant proteins using *Escherichia coli*. Appl Microbiol Biot.

[26] Marx CK, Hertel TC, Pietzsch M (2008). Random mutagenesis of a recombinant microbial transglutaminase for the generation of thermostable and heat-sensitive variants. J Biotechnol.

[27] Kieliszek M, Misiewicz A (2014). Microbial transglutaminase and its application in the food industry. A review. Folia Microbiol.

[28] Marx CK, Hertel TC, Pietzsch M (2007). Soluble expression of a pro-transglutaminase from Streptomyces mobaraensis in Escherichia coli. Enzym Microb Technol.

[29] Sommer C, Volk N, Pietzsch M (2011). Model based optimization of the fed-batch production of a highly active transglutaminase variant in *Escherichia coli*. Protein Expr Purif.

[30] Oteng-Pabi SK, Keillor JW (2013). Continuous enzyme-coupled assay for microbial transglutaminase activity. Anal Biochem.

[31] Zhang L, Zhang L, Yi H, Du M, Ma C, Han X, Feng Z, Jiao Y, Zhang Y (2012). Enzymatic characterization of transglutaminase from *Streptomyces mobaraensis* DSM 40587 in high salt and effect of enzymatic cross-linking of yak milk proteins on functional properties of stirred yogurt. J Dairy Sci.

[32] Marx CK, Hertel TC, Pietzsch M (2008). Purification and activation of a recombinant histidine-tagged pro-transglutaminase after soluble expression in *Escherichia coli* and partial characterization of the active enzyme. Enzyme Microb Tech.

[33] Folk JE, Cole PW (1966). Mechanism of action of guinea pig liver transglutaminase. I. Purification and properties of the enzyme: identification of a functional cysteine essential for activity. J Biol Chem.

[34] Strutz W. Exploring Protein Stability by NanoDSF. Biophys J. 2016;110:393a.

[35] Buettner K, Hertel TC, Pietzsch M (2012). Increased thermostability of microbial transglutaminase by combination of several hot spots evolved by random and saturation mutagenesis. Amino Acids.

[36] Grodowska K, Parczewski A (2010). Organic solvents in the pharmaceutical industry. Acta Pol Pharm.

[37] Vigo TL. Textile processing and properties: Preparation, dyeing, finishing and performance. Amsterdam: Elsevier; 2013.

[38] Wypych G (2014). Textile industry. Handbook of solvents.

[39] Fontana A, Spolaore B, Mero A, Veronese FM (2008). Site-specific modification and PEGylation of pharmaceutical proteins mediated by transglutaminase. Adv Drug Deliv Rev.

[40] Dennler P, Chiotellis A, Fischer E, Bregeon D, Belmant C, Gauthier L, Lhospice F, Romagne F, Schibli R (2014). Transglutaminase-based chemo-enzymatic conjugation approach yields homogeneous antibody-drug conjugates. Bioconjug Chem.

[41] Karanikas EK, Kosolia CT, Zarkogianni MC, Nikolaidis NF, Tsatsaroni EG (2013). Effect of enzymatic treatment on the dyeing properties of protein wool fibers. Fiber Polym.

[42] Taylor MM, Bumanlag L, Marmer WN, Brown EM (2006). Use of enzymatically modified gelatin and casein as fillers in leather processing. J Am Leather Chem As.

[43] Shuster V, Fishman A (2009). Isolation, cloning and characterization of a tyrosinase with improved activity in organic solvents from *Bacillus megaterium*. J Mol Microbiol Biotechnol.

[44] Serdakowski AL, Dordick JS (2008). Enzyme activation for organic solvents made easy. Trends Biotechnol.

[45] Griebenow K, Klibanov AM (1996). On protein denaturation in aqueous-organic mixtures but not in pure organic solvents. J Am Chem Soc.

[46] Klibanov AM (2001). Improving enzymes by using them in organic solvents. Nature.

[47] Buchert J, Ercili Cura D, Ma H, Gasparetti C, Monogioudi E, Faccio G, Mattinen M, Boer H, Partanen R, Selinheimo E (2010). Crosslinking food proteins for improved functionality. Annu Rev Food Sci Technol.

[48] Heck T, Faccio G, Richter M, Thony-Meyer L (2013). Enzyme-catalyzed protein crosslinking. Appl Microbiol Biotechnol.

[49] Tang CH, Yang M, Liu F, Chen Z (2013). A novel process to efficiently form transglutaminase-set soy protein isolate-stabilized emulsion gels. Lwt-Food Sci Technol.

[50] Tang CH, Wu H, Chen Z, Yang XQ (2006). Formation and properties of glycinin-rich and beta-conglycinin-rich soy protein isolate gels induced by microbial transglutaminase. Food Res Int.

[51] Laemmli UK (1970). Cleavage of structural proteins during the assembly of the head of bacteriophage T4. Nature.

